# SARS-CoV-2 and *Streptococcus pneumoniae* Coinfection in a Previously Healthy Child

**DOI:** 10.1155/2021/8907944

**Published:** 2021-12-08

**Authors:** Kimberly C. Vu, Gloria P. Heresi, Michael L. Chang

**Affiliations:** Children's Memorial Hermann Hospital, The University of Texas, Health Science Center McGovern Medical School, Houston, TX 77030, USA

## Abstract

Coronavirus disease 2019 (COVID-19), caused by severe acute respiratory syndrome coronavirus 2 (SARS-CoV-2), was first reported in December 2019 in Wuhan, China. This novel coronavirus has been responsible for a pandemic that continues to devastate nations worldwide. COVID-19, like other viruses, causes pneumonia. However, unlike other viral respiratory tract infections such as influenza, bacterial coinfection in COVID-19 patients has uncommonly been described in adult and pediatric patients. We report a case of *Streptococcus pneumoniae* and COVID-19 coinfection in a previously healthy 4-year-old child.

## 1. Introduction

SARS-CoV-2 is responsible for the current global pandemic of COVID-19. As of June 2021, in the United States alone, there have been over 32,000,000 confirmed cases and over 550,000 deaths. However, compared to adults, pediatric patients have been far less impacted thus far. According to the CDC, laboratory-confirmed cases for children <18 years comprise 12–14% of the case burden, although representing about 22% of the US population. Infected children are most commonly asymptomatic or have mild symptoms, with the actual number probably underestimated.

Coinfection with bacterial pathogens is commonly identified with viral respiratory infections such as influenza. A low rate of bacterial coinfections has been reported with COVID-19, 1–4% rate in multiple adult studies [1–4]. In children, there are scarce reports only and there is little information regarding bacterial coinfection with SARS-CoV-2.

Here, we report a previously healthy and vaccinated child with COVID-19 and *Streptococcus pneumoniae* bacterial coinfection.

## 2. Case Presentation

**Figure 1:**
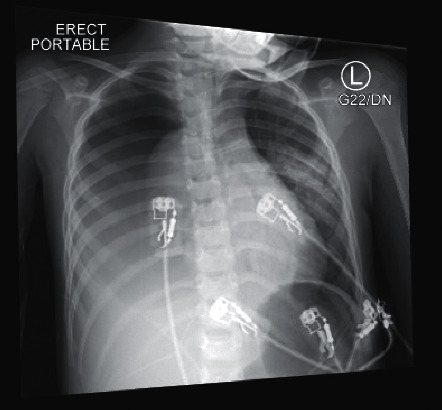
Large right pneumothorax; left perihilar and retrocardiac opacities with cystic changes concerning for infection; right pleural effusion.

**Figure 2:**
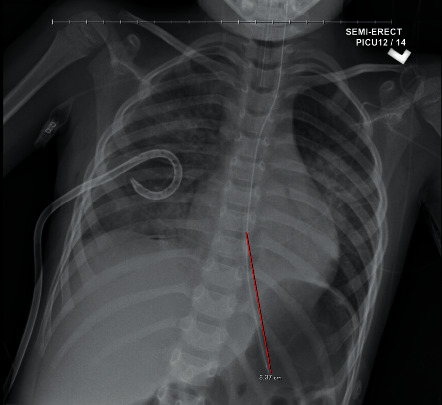
An endotracheal tube with the tip projecting over the mid-thoracic trachea; well-inflated lungs with multifocal patchy opacities likely compatible with multifocal infection, hemorrhage, ARDS, and/or pulmonary edema; small right hydropneumothorax with the chest tube in place.

A previously healthy fully vaccinated 4-year-old boy presented to the emergency department (ED) with a two-week history of fever, cough, and worsening shortness of breath. The patient presented to an outpatient clinic at the onset of symptoms on April 12, 2021, and tested positive for SARS-CoV-2 by PCR via nasopharyngeal swab. His symptoms continued to worsen with nasal flaring, tachypnea, and subcostal retractions prompting his return to the ED nine days after his positive COVID-19 test. In the ED, he had a temperature of 99.9°F, a heart rate of 142 beats per minute, a respiratory rate of 26 breaths per min, and 82% oxygen saturation in room air. The patient received supplemental oxygen via a non-rebreather mask. SARS-CoV-2 and influenza A/B nasopharyngeal swab specimens were sent for RT-PCR testing, positive only for SARS-CoV-2. On physical exam, the patient had increased work of breathing and diminished breath sounds on the right side. Blood and urine cultures were obtained. Cefepime and vancomycin antibiotic therapy were initiated. Blood work revealed significant leukocytosis with WBC of 21.2 K/uL, 57% neutrophils, 23% lymphocytes, and increased metamyelocytes at 6%. Chest radiograph (Figure 1) revealed a large right-sided pneumothorax and pleural effusion. A chest tube drained 250 mL of purulent fluid (Figure 2). Pleural fluid analysis was consistent with empyema: pH 6.5, WBC 187,590/uL, glucose 3.0 mg/dL, protein 5.0 g/dL, and LDH 37211 IU/L. Initial Gram staining showed Gram-positive cocci in chains. The patient continued to deteriorate, requiring intubation and transfer to the PICU.

On hospital day #2, the patient was extubated and transitioned to a high-flow nasal cannula. Culture of the pleural fluid isolated *S. pneumoniae* (penicillin MIC 0.023 ug/mL). Unfortunately, the pneumococcal isolate was not sent for serotyping. Cefepime and vancomycin were de-escalated to ceftriaxone. Over the next eleven days, the patient weaned to room air and the chest tube was removed. He was transitioned to oral amoxicillin and discharged home. The patient was seen two weeks after discharge and appeared well with no further cough or respiratory complaints. Parents reported some fatigue with exertion initially upon discharge from the hospital, but that continued to improve.

## 3. Discussion

We report a previously healthy, fully vaccinated 4-year-old child with no known risk factors to develop severe acute respiratory COVID-19 who had severe SARS-CoV-2 infection complicated by *S. pneumoniae* coinfection. It is unclear how the codetection of SARS-CoV-2 and *S. pneumoniae* impacted the patient's disease severity and clinical course. The patient had more severe disease than would be expected in a pediatric patient with acute SARS-CoV-2 infection, although the clinical course could be consistent with pneumococcal pneumonia alone.

COVID-19 has impacted children less compared to the adult population. A majority of pediatric patients are asymptomatic or have milder cases. According to the American Academy of Pediatrics (AAP) State-Level Data Report, the pediatric population made up 1.4%–3.2% of total reported hospitalizations. Only between 0.1% and 1.9% of all children with COVID-19 cases resulted in hospitalizations. Of all COVID-19 deaths and all child COVID-19-related deaths, children only made up 0.00%–0.23% and 0.00%–0.03%, respectively.

Commonly identified risk factors for hospitalizations in pediatric patients included obesity, chronic lung disease, and prematurity with gestational age <37 weeks [5]. Our patient had none of these risk factors, making the patient's severe clinical course even more surprising. A systematic review of imaging findings of COVID-19 in 431 children also showed that pleural effusions were extremely rare, with only three total cases in that study [6].

Bacterial copathogens are commonly identified in viral respiratory tract infections such as influenza—usually, *S. pneumoniae* or *Staphylococcus aureus*. Rates of coinfection in severe flu have been reported as high as 20–30%. In multiple studies of children with RSV, bacterial coinfection rates ranged 40–50% [7–10]. The rate of bacterial coinfection in patients with COVID-19 has been relatively rare and uncommon. Few published studies report rates of only 1.2 to 3.5% of bacterial coinfections [1–4]. A meta-analysis by Lansbury et al. identified *Mycoplasma pneumoniae,* followed by *Pseudomonas aeruginosa, Haemophilus influenzae*, and *Klebsiella pneumoniae*, only one case of methicillin-resistant *S. aureus* coinfection, and no patients with coinfection with *S. pneumoniae* or *Streptococcus pyogenes* [3]. In studies conducted in pediatric patients with COVID-19 in Wuhan, China, *M. pneumoniae* was the most commonly identified bacterial pathogen, with rates ranging from 12% up to 37.3% [11]. Only 0.025% of patients were identified with the invasive pneumococcal disease in confirmed SARS-CoV-2 infection in adults, with none in children [12]. In one multicenter Italian study with 168 children with COVID-19, there was only one case of bacterial coinfection with *S. pneumoniae* [13].

As previously seen in other viral respiratory tract infections, the presence of a bacterial coinfection is typically associated with an increase in severity and frequent cause of mortality. However, with such limited data of bacterial coinfection in patients with COVID-19, it has been difficult to characterize its impact on the patient's clinical course fully. A postmortem study in people with COVID-19 demonstrates that about 32% of the patients had a histopathology consistent with bacterial lung superinfections. However, lung superinfections were found as the cause of death in only 16% of the patients with possible bacterial infection and only 3% of all patients within the study who had a postmortem exam [14]. Another review identified a study showing a significant difference in COVID-19 patients with a bacterial coinfection compared to patients without coinfection, with an increase in length of hospital stay and in-hospital mortality [15].

In fact, due to the social distancing, handwashing, and masking protocols implemented during the COVID-19 pandemic, there has been a significant decrease in the incidence of pediatric respiratory infections. Studies from multiple countries showed more than 50% decrease in respiratory tract-related infection emergency department visits and a much shorter influenza and RSV season than previous years with a dramatic reduction in hospitalizations due to non-COVID-19 respiratory illnesses [16–19]. A prospective analysis of surveillance data from 26 countries and territories estimated a 38% decrease in the incidence of reported *S. pneumoniae* invasive infections during the COVID-19 pandemic. As the study continued, there was an additional average 13% weekly reduction of reported cases of *S. pneumoniae*. Similar results were found for the incidence of invasive disease caused by *H. influenza* and *N. meningitidis* [20]. With such findings, it is unlikely that our patient with COVID-19 had two unrelated infections, given a significant decrease in pneumococcal disease alone.

To our knowledge, this is one of the two reported cases of pneumococcal pneumonia superinfection with acute COVID-19 disease in a pediatric patient. The reason for a low rate of coinfection is unclear, and empiric antibiotic therapy is probably not necessary for most pediatric patients with COVID-19 respiratory tract infections. However, providers should be suspicious of bacterial coinfection in pediatric patients with unusually severe presentations or uncommon radiographic findings of what is assumed to be acute SARS-CoV-2 respiratory tract infection.

## Data Availability

The data used to support the findings of this study are included within the article.
